# Nuclear Envelope Retention of LINC Complexes Is Promoted by SUN-1 Oligomerization in the *Caenorhabditis elegans* Germ Line

**DOI:** 10.1534/genetics.116.188094

**Published:** 2016-04-18

**Authors:** Anahita Daryabeigi, Alexander Woglar, Antoine Baudrimont, Nicola Silva, Dimitra Paouneskou, Cornelia Vesely, Manuel Rauter, Alexandra Penkner, Michael Jantsch, Verena Jantsch

**Affiliations:** * Department of Chromosome Biology, Max F. Perutz Laboratories, Vienna Biocenter, University of Vienna, 1030, Austria; † Center for Anatomy and Cell Biology, Department of Cell and Developmental Biology, Medical University of Vienna, 1090, Austria

**Keywords:** oocyte formation, meiosis, LINC complex, *Caenorhabditis elegans*

## Abstract

SUN (Sad1 and UNC-84) and KASH (Klarsicht, ANC-1, and Syne homology) proteins are constituents of the inner and outer nuclear membranes. They interact in the perinuclear space via C-terminal SUN-KASH domains to form the linker of nucleoskeleton and cytoskeleton (LINC) complex thereby bridging the nuclear envelope. LINC complexes mediate numerous biological processes by connecting chromatin with the cytoplasmic force-generating machinery. Here we show that the coiled-coil domains of SUN-1 are required for oligomerization and retention of the protein in the nuclear envelope, especially at later stages of female gametogenesis. Consistently, deletion of the coiled-coil domain makes SUN-1 sensitive to unilateral force exposure across the nuclear membrane. Premature loss of SUN-1 from the nuclear envelope leads to embryonic death due to loss of centrosome–nuclear envelope attachment. However, in contrast to previous notions we can show that the coiled-coil domain is dispensable for functional LINC complex formation, exemplified by successful chromosome sorting and synapsis in meiotic prophase I in its absence.

IN eukaryotic cells, the nuclear envelope (NE) forms a barrier between nuclear contents and the cytoplasm. It consists of the inner nuclear membrane (INM) and outer nuclear membrane (ONM), which are connected with the ER. Linker of nucleoskeleton and cytoskeleton (LINC) complexes reside in the NE and form the main connection between the cytoplasm and nucleus. These complexes comprise two conserved protein families: SUN (sad1 and UNC-84) and KASH (Klarsicht, ANC-1 and SYNE homology). KASH proteins are found at the ONM, whereas SUN proteins reside at the INM. Almost all SUN proteins contain at least one transmembrane domain, coiled-coil (cc) motifs, and a conserved SUN domain. The cc and SUN domains reside in the perinuclear space and are required for interaction with KASH proteins (Chang *et al.* 2015 for review). The cytoplasmic region of KASH-domain proteins can interact with various cytoskeletal elements and associated motor proteins. The N-terminals of SUN-domain proteins reach into the nucleus, where they interact with both chromatin and the nuclear lamina (for review, see Fridkin *et al.* 2009; Razafsky and Hodzic 2009; and Starr and Fridolfsson 2010). The SUN-KASH bridge supports numerous biological processes, including centrosome positioning to the NE and nuclear migration (Malone *et al.* 1999, 2003; X. Zhang *et al.* 2009; Schneider *et al.* 2011), or transduction of cytoplasmic forces during meiotic chromosome movement (Fridkin *et al.* 2009; Hiraoka and Dernburg 2009).

*Caenorhabditis elegans* Matefin/SUN-1 is expressed throughout the germ line and in early embryos (Malone *et al.* 2003; Fridkin *et al.* 2004; Penkner *et al.* 2007). The *C. elegans* germ line therefore provides a tissue in which SUN-1 structure/function studies can be readily followed by a well-defined biological readout. The germ line comprises a syncytium in which mitotically-proliferating nuclei at the distal portion give rise to meiocytes. These enter meiosis and go through the prolonged prophase of the first meiotic division (Greenstein 2005). At the gonad bend, meiocytes undergo cellularization to form oocytes. Once they pass the spermatheca they are fertilized and immediately undergo two meiotic divisions (Hubbard and Greenstein 2005).

Successful gamete formation requires proper segregation of the parental homologous chromosomes in the first meiotic division. To achieve this with high accuracy, homologous chromosomes must move, pair, and recombine during prophase (Gerton and Hawley 2005). Movement is achieved by transmitting microtubule-mediated forces to chromosome ends via the SUN-1-ZYG-12 (KASH homolog expressed in *C. elegans* germ line) NE bridge (Baudrimont *et al.* 2010; Wynne *et al.* 2012; Labrador *et al.* 2013; Woglar and Jantsch 2014). Interfering with force transmission leads to a lack of presynaptic chromosome alignment and synaptonemal complex (SC) establishment between nonhomologs (Penkner *et al.* 2007; Sato *et al.* 2009; Labrador *et al.* 2013). In mitotic cells SUN-1 is evenly distributed along the NE. During the chromosome movement stage, corresponding to leptotene/zygotene and also known as the transition zone (TZ) in *C. elegans*; SUN-1 relocates to form pronounced aggregates around chromosome ends in close proximity to the NE (Penkner *et al.* 2009; Sato *et al.* 2009), a feature conserved in vertebrates (Ding *et al.* 2007; Schmitt *et al.* 2007), which is mirrored by ZYG-12 aggregates (Labrador *et al.* 2013; Sato *et al.* 2009). Aggregate formation and consequent chromosome end mobilization are seemingly controlled by signals from inside the nucleus involving the *chk-2* and polo kinases (PLKs 1 and 2) (Penkner *et al.* 2009; Harper *et al.* 2011; Labella *et al.* 2011).

*sun-1* null mutants are sterile and gonads degenerate with decreased numbers of irregularly-sized aneuploid nuclei (Fridkin *et al.* 2004; Penkner *et al.* 2007). The LINC complex also contributes to gonad architecture by ZYG-12-mediated recruitment of dynein at the NE to build up the tension required for nuclear positioning (K. Zhou *et al.* 2009). In embryos this complex mediates centrosome attachment to the NE (Malone *et al.* 2003).

The role of the cc motifs in the oligomeric nature of SUN proteins has been put forward in several studies (Padmakumar *et al.* 2005; Crisp *et al.* 2006; Q. Wang *et al.* 2006, 2012; Lu *et al.* 2008; Sosa *et al.* 2012; Z. Zhou *et al.* 2012). The structure of the mammalian SUN-domain protein SUN2 has been elucidated in the context of binding to the KASH partner Nesprin (Sosa *et al.* 2012; W. Wang *et al.* 2012; Z. Zhou *et al.* 2012). SUN2 forms trimers mediated by the region adjacent to the SUN domain, which forms a helical stem to create a clover-like structure. KASH peptides reside between the trimeric SUN domains and multiple hydrophobic interactions mediate KASH-peptide binding to SUN-domain interfaces (Figure 1A). Measurements have shown that the triple-helical cc fits into the perinuclear space (Sosa *et al.* 2012), suggesting that the LINC complex, and the SUN-protein cc domain in particular, have a role in maintaining an even spacing. Interestingly, in *C. elegans* the UNC-84 protein can be mutated without affecting nuclear membrane spacing, except in body wall muscle cells where nuclei are under mechanical stress (Cain and Starr 2015). The structural analysis of SUN-KASH peptides led to the development of models for higher-order assembly of the SUN-KASH module. The SUN trimers may bind to multiple KASH peptides from several different KASH oligomers to build up higher-order clusters of the LINC complex (Sosa *et al.* 2012).

**Figure 1:**
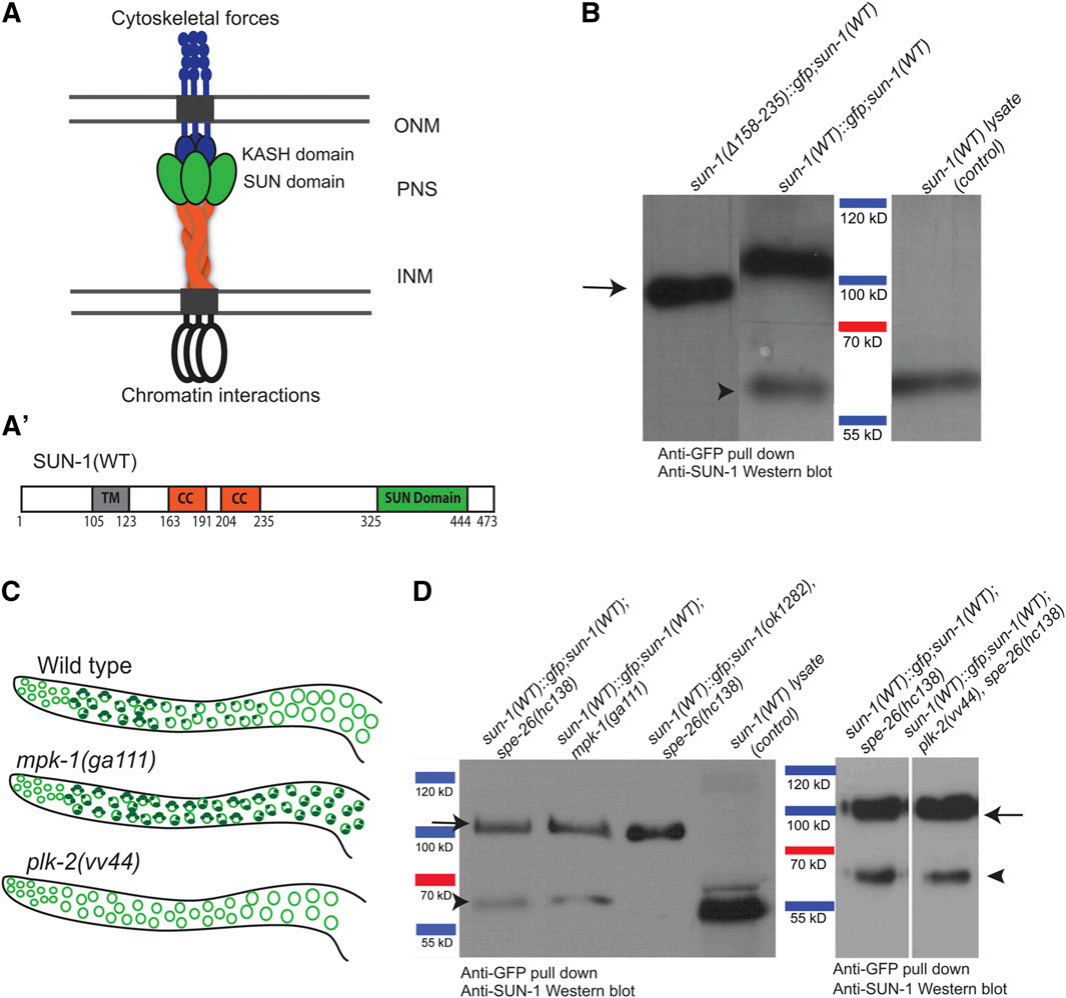
SUN-1 oligomerization is disrupted in the absence of cc regions. (A) Current model of the nuclear membrane-spanning LINC complex based on structural data from mammalian SUN2 (see *Introduction*). SUN-protein trimers recruit three KASH proteins and thereby form a (hetero) hexameric complex. INM, inner nuclear membrane; ONM, outer nuclear membrane; PNS, perinuclear space. (A’) Schematic drawing of the domains of wild-type SUN-1. CC, coiled-coil domains; TM, transmembrane domain. (B) Western blot of GFP pull-downs in wild type and the SUN-1(Δ158–235) mutant. Arrow, the GFP-tagged SUN-1 protein, ∼100 kDa; arrowhead, endogenous SUN-1, 60 kDa. The latter band is missing when the cc regions are deleted. (C) Schematic diagrams of SUN-1 distribution in different mutant backgrounds and wild type. (D) Western blot of GFP pull-down in wild type and different mutant backgrounds. Arrow, 100-kDa SUN-1(WT)::GFP: band; arrowhead, 60-kDa endogenous SUN-1 band. SUN-1 self-interaction occurs at all stages of meiosis. SUN-1(WT) lysate was used as a control to identify the endogenous SUN-1 protein band.

Previous yeast two-hybrid (Y2H) assays proposed a role for *C. elegans* SUN-1-predicted cc regions in self-interaction (Minn *et al.* 2009). To investigate the function of these domains in more detail, we generated several mutants with deletions in the cc regions and assessed the phenotype and cellular readout of the mutations in the germ line and embryos. Here, we show that despite disrupting SUN-1 oligomerization, deletion of the cc domains does not abrogate the formation of a functional LINC complex. Surprisingly, all aspects of SUN-1-mediated chromosome pairing and synapsis do not rely on the SUN-1 oligomerization domains, albeit chromosome movement exerts mechanical strain on the LINC complex. Strikingly, the cc region has a role in efficient protein retention at the NE, which becomes more pronounced prior to oocyte cellularization in the germ line.

## Materials and Methods

### C. elegans strains and maintenance

All worm strains were maintained using standard techniques (Brenner 1974). A complete list of strains used in this study is reported in Supplemental Material, Table S1. The following mutations were used in this study: LGI: *plk-2(vv44)*; LGIII: *mpk-1(ga111)*, *unc-79(e1068)*, *unc-119(ed3)*; LGIV: *spe-26(hc138)*, *ced-3(n717)*; and LGV: *sun-1(ok1282)*.

The following transgene insertions were used: jfSi1[Psun-1::GFP Cbr-unc-119(+)] II, jfSi7[Psun-1::DENDRA2 Cbr-unc-119(+)] II, jfSi34[Psun-1(∆158–235)::GFP Cbr-unc-119(+)] II, jfSi45[Psun-1(∆158–235) Cbr-unc-119(+)] II, jfSi63[Psun-1(∆158–235)::EOS3.2 Cbr-unc-119(+)] II, and ojIs9 [zyg-12all::GFP unc-119(+)]. The rearrangement used in this study was *nT1[qIs51]*(IV;V).

### Y2H assay

The split-ubiquitin based Y2H membrane protein system (MoBiTec P01001DS) was used for Y2H assays.

### Immunoprecipitation analysis

Worms were harvested in homogenization buffer and frozen at −80°. Worms were ground in liquid nitrogen and sonicated three times on ice (30 sec, amplitude of 70–80%). Protein lysates were centrifuged and supernatants were added to GFP-Trap agarose beads (ChromoTek) and incubated overnight at 4°. Beads were washed three times in wash buffer and boiled in SDS sample buffer at 90° for 10 min. Bound proteins were analyzed by SDS–PAGE.

To detect SUN-1, an antibody against either an N-terminal (guinea pig anti-SUN-1, EurogenTec; 1:3000; Penkner *et al.* 2009) or C-terminal segment (rabbit anti-SUN-1, EurogenTec; 1:500; Fridkin *et al.* 2004) was used. The secondary antibody was either HRP-conjugated anti-guinea pig (#6771, Abcam) or alkaline phosphatase-conjugated anti-guinea pig (#A5062; Sigma Chemical, St. Louis, MO) used at a 1:5000 dilution.

### Western blot analysis

Two hundred very young adult worms (containing at the most one to two eggs) were picked into lysis buffer (1× TE; 2× complete protease inhibitor) and snap frozen three times in liquid nitrogen. After the last thawing step, Laemmli buffer was added to a final concentration of 1× and worms were boiled for 10 min. Protein extracts were resolved on an acrylamide gel and transferred for 1 hr at 4°. Anti-SUN-1 (Penkner *et al.* 2009) and anti-actin (Santa Cruz Biotechnology) antibodies were both diluted to 1:3000 in 1× TBST (1× TBS, 0.1% Tween-20) to probe the membranes.

### Immunofluorescence analysis

L4 hermaphrodites were incubated at 20° for 24 hr. Gonads were dissected in PBS and fixed in 1% formaldehyde for 5 min (Martinez-Perez and Villeneuve 2005). For immunostaining of gonads, nonspecific binding sites were blocked with 3% BSA in PBS for 20 min. All antibodies were diluted in 3% BSA in PBS. Gonads were incubated with primary antibody overnight at 4° and with secondary antibody for 2 hr at room temperature.

Primary antibodies used were anti-GFP (#11 814 460 001, Roche; 1:500), anti-SUN-1 (EurogenTec; 1:300; Penkner *et al.* 2009), anti-HIM-8 (#0011645, Novus; 1:10,000), anti-SYP-1 (1:200; MacQueen *et al.* 2002), anti-HTP-3 (1:500; Goodyer *et al.* 2008), anti-RAD-51(1:250; Colaiacovo *et al.* 2003), anti-ZYG-12 (1:400; Malone *et al.* 2003), anti-phospho-SUN-1S8 (1:700; Penkner *et al.* 2009), and anti-SPD-5 (1:700; Dammermann *et al.* 2004). Secondary antibodies used were anti-mouse Alexa488, anti-guinea pig Alexa488, anti-rabbit Alexa568 and anti-rat Alexa568 [all Invitrogen (Carlsbad, CA) or Molecular Probes (Eugene, OR), all at 1:500 dilution].

### Fluorescence microscopy

All microscopy evaluations were done using a DeltaVision microscope with SoftWoRx image analysis deconvolution software (Applied Precision), ImageJ (National Institutes of Health), and Adobe Photoshop software. Intensity measurement of SUN-1::GFP in the nuclear rim of the mutant and wild-type animals was performed on non-deconvolved images. The average signal intensity of the rim and the average background for each nucleus were measured using ImageJ, and the GFP signal intensity was calculated by subtracting the mean fluorescence intensity of both.

### Live imaging

Live imaging was performed as described by (Baudrimont *et al.* 2010) and analysis was done with ImageJ using the stackreg and manual tracking plugins.

### Photo-conversion

To perform photo-conversion on *sun-1(∆158–235)*::*eos3.2*, young adult hermaphrodites were mounted in PBS containing 10 mM levamisole. The experiment was done using a DeltaVision deconvolution microscope (Applied Precision) with a DAPI filter, 100% laser intensity, and a 60× objective. Images of dissected gonads were acquired 4 hr postrecovery. Photo-conversion experiments on wild-type hermaphrodites tagged with Dendra2 were done with an LSM5 microscope (Carl Zeiss, Thornwood, NY) and were performed using a 405 nm filter with 100% laser output. A few cell rows of each zone were photo-converted and images were acquired after 8 hr, when photo-converted nuclei had reached the next meiotic stage.

To study SUN-1 *de novo* synthesis in embryos, young *sun-1(wt)*::*dendra2* hermaphrodite adults were mounted in PBS containing 10 mM levamisole and −1 diakinesis oocytes were bleached using a DeltaVision deconvolution microscope with a DAPI filter and 100% laser intensity. Images were acquired 90 min postrecovery.

### Deep sequencing

RNA was extracted from adult *Psun-1(wt)*::*gfp II*; *spe-26(hc138) IV*; *sun-1(ok1282) V* and *Psun-1(Δ158–235)*::*gfp II*; *spe-26(hc138) IV*; *sun-1(ok1282) V* hermaphrodites using TriFast (Peqlab, Erlangen, Germany) and Ribo-Zero magnetic kits, and fragmented and cleaned using ReliaPrep RNA minicolumns (Promega, Madison, USA). After complementary DNA synthesis, samples were sequenced. Quantification of the complete library was done using an Agilent Technologies Bioanalyzer DNA 1000 assay kit and a qPCR NGS library quantification kit (Agilent Technologies). Cluster generation and sequencing was carried out using the Illumina Genome Analyzer IIx system. After sequencing at a read length of 36 bp, adaptor sequences were removed using Cutadapt (http://code.google.com/p/cutadapt/).

### RNA interference

RNA interference (RNAi) feeding was performed with the *zyg-12* clone from the Ahringer library as described in (Kamath *et al.* 2001). L4 worms were incubated at 20° for 48 hr before dissection.

### Data availability

Strains are available upon request. Sequence data are available at GEO under the submission number GSE76773. The authors state that all data necessary for confirming the conclusions presented in the article are represented fully within the article.

## Results

### SUN-1-predicted cc domains are required for self-interaction

*C. elegans* SUN-1 contains two predicted cc motifs, one spanning residues 163–191 (cc1) and the second spanning residues 204–235 (cc2) (Figure 1A’). In agreement with Minn *et al.* (2009), we confirmed that SUN-1 self-interaction in a Y2H assay was lost following combined deletion of cc1 and cc2 (∆158–235) (Figure S1A). Deleting individual cc1 and cc2 domains weakened or disrupted self-interaction (our unpublished results).

To study the importance of the cc motifs in SUN-1 oligomerization, we performed co-immunoprecipitation (coIP) analysis of GFP-tagged single-copy integrated *sun-1* wild type [SUN-1(WT)::GFP, a functional transgene described in Woglar *et al.* (2013)], or mutants with deleted cc motifs [SUN-1(∆158–235)::GFP] (Frokjaer-Jensen *et al.* 2008). The analysis was performed in the presence of endogenous wild-type SUN-1.

SUN-1(WT)::GFP coIP and the subsequent Western blot analysis revealed 2 bands of 100 and 60 kDa, corresponding to the GFP-tagged transgene and coprecipitated endogenous SUN-1. In the absence of the cc motifs, we could not detect a band corresponding to coprecipitated endogenous SUN-1. This result shows that the transgenic-tagged protein cannot interact with wild-type protein in the absence of its luminal cc domain (Figure 1B).

SUN-1 is evenly distributed throughout the NE during late meiotic prophase and mitotic interphase. In contrast, during early meiotic prophase and mitotic metaphase/anaphase SUN-1 forms aggregates at chromosome ends or spindle poles (Penkner *et al.* 2007; Penkner *et al.* 2009). We next investigated whether differences in the SUN-1 localization pattern (as aggregates or evenly distributed throughout the NE) correlated with cc-driven SUN-1 oligomerization in the germ line. To address this, we co-immunoprecipitated SUN-1(WT)::GFP/SUN-1(WT) in previously-described mutant backgrounds either enriched for or lacking SUN-1 aggregates. *mpk-1(ga111)* mutants arrest in leptotene/zygotene at restrictive temperature, and their gonads are therefore highly enriched in SUN-1 aggregates at chromosome end attachments (Figure 1C and Figure S1B). These mutants do not produce embryos (Lackner and Kim 1998; Leacock and Reinke 2006; Lee *et al.* 2007; Arur *et al.* 2009). In contrast, SUN-1 fails to form aggregates in the TZ in *plk-2(vv44)* gonads, and within the germ line SUN-1 is evenly distributed throughout the NE (Figure 1C) (Labella *et al.* 2011). Here we used the fertilization-defective background *spe-26(hc138)* to prevent the presence of embryos in our extracts (Varkey *et al.* 1995). In both mutant backgrounds we could detect the coprecipitated endogenous SUN-1 protein as in the wild type (Figure 1D).

These data suggest that SUN-1 forms oligomers in all populations of SUN-1 (both at the rim and the aggregates at chromosome ends) and that oligomerization is only abrogated in the absence of the cc motifs.

### Deletion of cc domains leads to loss of SUN-1 from the NE, particularly in late prophase I

To elucidate the function of SUN-1 oligomerization, we analyzed SUN-1 localization in the germ line, brood size, offspring viability, and the occurrence of male offspring (as an indicator of meiotic chromosome nondisjunction) of self-fertilized hermaphrodites expressing a single-copy integration of either untagged or GFP-tagged *sun-1(*Δ*158–235)* constructs in the absence of endogenous SUN-1 [*sun-1(ok1282)*]. All experiments were performed in both tagged and untagged mutant hermaphrodites, unless mentioned otherwise.

**Figure 2:**
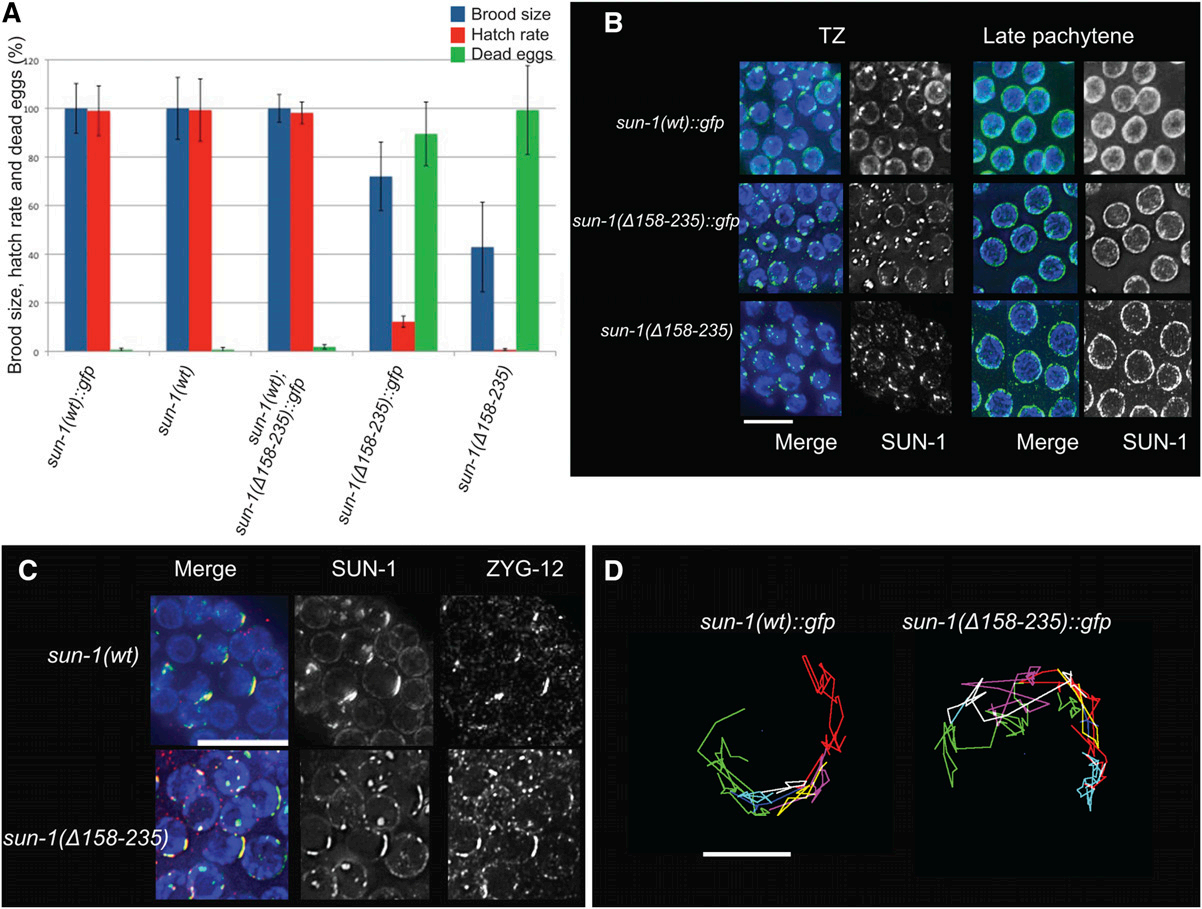
Deletion of the cc motifs reduces embryo viability but does not affect formation of a functional LINC complex in early prophase I. (A) Brood size, hatch rate, and the number of dead eggs for each genotype were counted throughout the life span of the hermaphrodite worm *Psun-1(wt)*::*gfp*; *sun-1(ok1282) V*, *n* = 14; *Psun-1(*Δ *158-235)*::*gfp II*; *sun-1(ok1282) V*, *n* = 13; *sun-1(wt)*, *n* = 10; *Psun-1(*Δ*158-235) II*; *sun-1(ok1282) V*, *n* = 10. The brood size of mutants was normalized to that of the wild type (100%). Bars represent SEM. The drop in brood size and hatch rate is significant in both mutants (two-tailed *t*-test, *P* < 0.0001). (B) Magnified images showing SUN-1 distribution in wild type and mutants lacking both cc regions (DAPI, blue; SUN-1, green). Note the formation of SUN-1 aggregates in the TZ of all genotypes. Bar 10 µm. (C) DAPI (blue), SUN-1 (green), and ZYG-12 (red) staining shows ZYG-12 recruitment to SUN-1 aggregates in the TZ in the wild type and mutant. Bar, 10 µm. (D) Representative displacement tracks of SUN-1 aggregates representing chromosome end movement over 3 min in wild-type and mutant nuclei. Wild type *n* = 9 and mutant *n* = 14 nuclei. Bar, 2 µm.

Deletion of SUN-1 cc motifs had a severe impact on offspring viability: the brood size of *sun-1(*Δ*158–235)*::*gfp* was 72% of the wild type and only 12% of the eggs hatched. Brood size and hatch rate were even more dramatically decreased in the *sun-1(*Δ*158–235)* untagged mutant: the brood size was 50% of wild type and only 0.7% of eggs gave rise to viable progeny (Figure 2A). These results suggest that the propensity of GFP to dimerize mitigates the effect of deletion of the cc domains. Subsequent analyses were consistent with the GFP-tag mutant being a hypomorphic allele. Analysis of offspring viability in the presence of endogenous wild-type protein revealed that the mutant is fully recessive to the wild type (Figure 2A). In contrast to hermaphrodites that carry two *X* chromosomes, males have only one *X* chromosome and arise by spontaneous meiotic nondisjunction of the *X* chromosome. An increased number of males (*i.e.*, a high incidence of males, him phenotype) therefore indicates a failure in meiotic pairing or recombination (Hodgkin *et al.* 1979). Among the surviving progeny of mutant hermaphrodites the ratio of males was not increased, reflecting the undisturbed segregation of the *X* chromosomes. To elucidate the cause of embryonic death (be it through aneuploidy or developmental defects), we investigated SUN-1 localization in the germ line and the processes of prophase of meiosis I in more detail in both the tagged and untagged mutant.

Abrogation of SUN-1 oligomerization did not impair SUN-1 reorganization into aggregates at the onset of meiosis. Immunofluorescence staining showed SUN-1 aggregates in the TZ and even distribution of the protein from midpachytene onward, as in wild type (Figure 2B). Against expectation, ZYG-12 was successfully recruited to the ONM when both cc domains were deleted (Figure 2C). Consistent with this observation, *in vivo* time-lapse imaging of SUN-1 aggregates in worms expressing either *sun-1(*Δ*158–235)*::*gfp* or *sun-1(WT)*::*gfp* as the sole source of SUN-1 revealed that deletion of these motifs had no effect on chromosome movement. Figure 2D shows the displacement tracks of SUN-1 aggregate in *sun-1(WT)*::*gfp II*; *sun-1(ok1282)V* and *sun-1(Δ158–235)*::*gfp II*; *sun-1(ok1282)V* over 3 min. The average velocity of SUN-1 aggregates was 52.73 nm/sec (*n* = 9 nuclei) in the wild type *vs.* 65.76 nm/sec (*n* = 14 nuclei) in the mutant. Movement of the aggregates in the mutant indicated the formation of a functional LINC complex and the successful transduction of cytoskeletal forces to chromosome ends.

**Figure 3:**
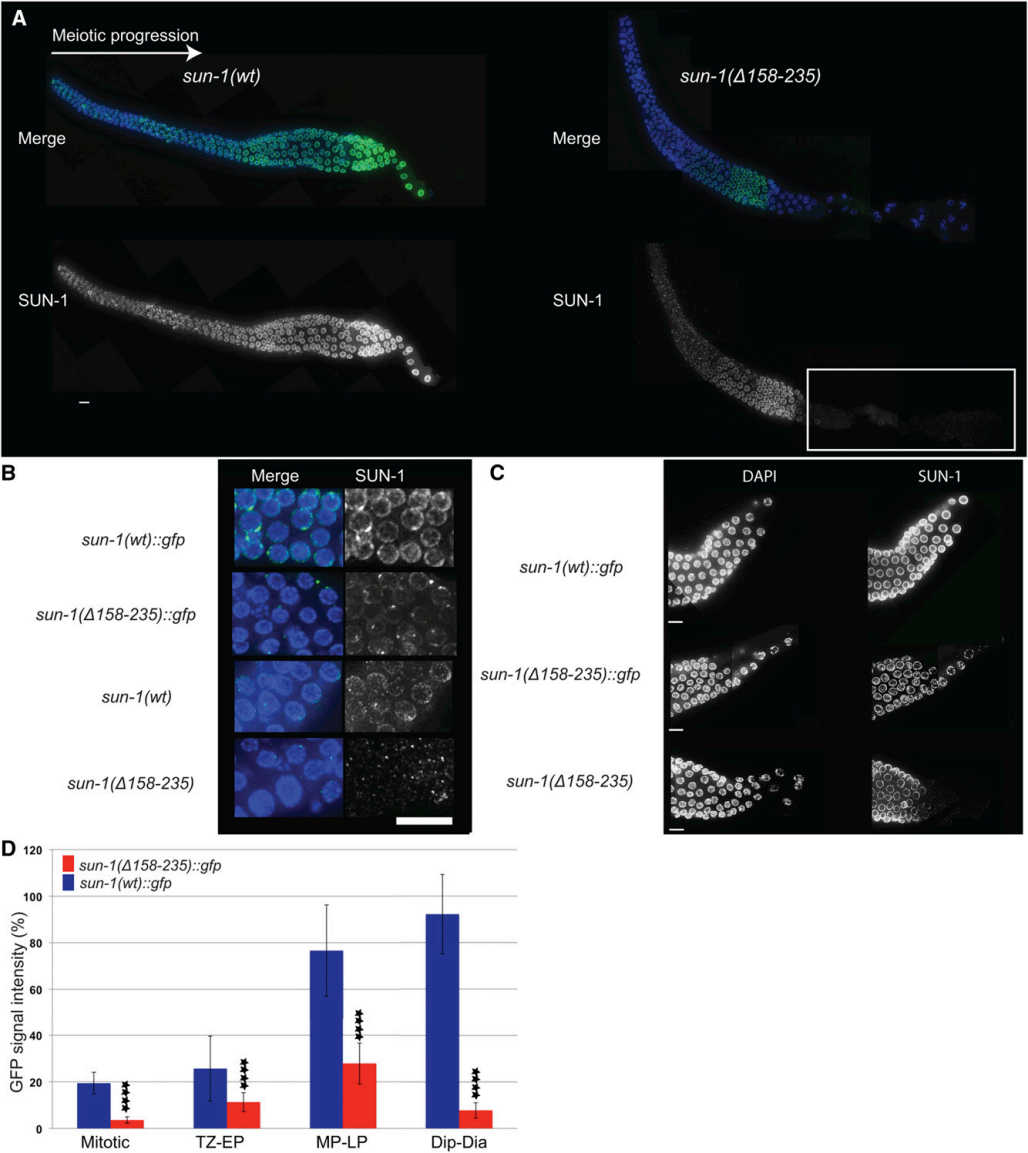
SUN-1 fails to maintain NE localization in the absence of cc motifs, especially in late prophase. (A) SUN-1 distribution in wild-type and *Psun-1(*Δ*158–235) II*; *sun-1(ok1282) V* mutant gonad. The white box indicates the zone where SUN-1 is barely detectable in the mutant. (B) DAPI (blue) and SUN-1/GFP (green) costaining in the mitotic zone of worms expressing tagged and untagged wild-type and mutant SUN-1 (in the absence of endogenous wild-type SUN-1). Note the reduced SUN-1 signal intensity in the rim of mitotic nuclei in the mutants. (C) Late pachytene and diplotene stages of *sun-1(wt)*::*gfp* and mutant gonads. Note the pronounced loss of SUN-1 from the NE in the mutants. Bar, 10 µm. (D) SUN-1::GFP signal intensity in the nuclear rim of the mutant and wild-type gonads. **** denotes the significant decreased amount of SUN-1 in the NE throughout the gonad (two-tailed *t*-test, *P* < 0.0001 for each zone). For each zone 30 nuclei of 3 independent gonads were analyzed. Error bars represent SD. The experiment was performed in the absence of endogenous wild-type SUN-1. Dia, diakinesis; Dip, diplotene; EP, early pachytene; LP, late pachytene; MP, midpachytene; TZ, transition zone.

When studying SUN-1 distribution we detected an overall decrease in SUN-1 signal intensity in the entire mutant germ line compared to wild type (Figure 3, A–D). This drop in signal intensity was most severe in the later stages of prophase I prior to oocyte cellularization; from this stage onwards, the SUN-1 signal was barely detectable (Figure 3, A, C, and D). We also noticed that the GFP tag slowed down the rate of SUN-1 signal loss slightly and thus represents a milder allele (Figure 3C).

**Figure 4:**
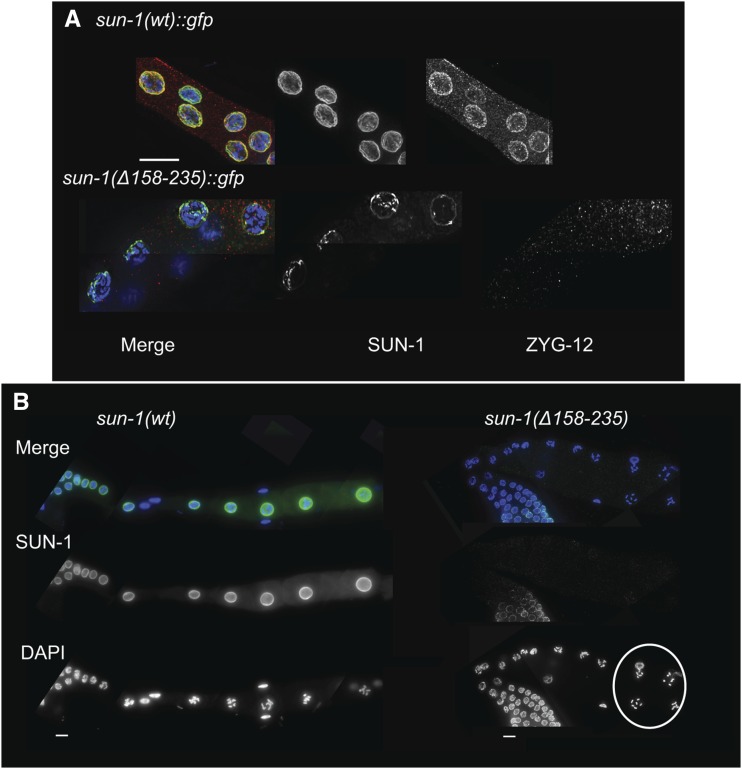
SUN-1 loss is most prominent at late stages of prophase I. (A) DAPI (blue), SUN-1 (green), and ZYG-12 (red) staining of diplotene nuclei in the wild type and *Psun-1(*Δ*158–235) II*; *sun-1(ok1282) V* mutant. Note the prominent loss of both SUN-1 and ZYG-12 in the later stages of meiosis in the mutant (32 out of 40 mutant nuclei had no ZYG-12 signal and 8 displayed barely detectable signal). (B) Loss of SUN-1 leads to misalignment of the oocytes. DAPI (blue) and SUN-1 (green) staining in wild-type and mutant germ lines. The white circle shows disruption of the wild-type alignment of oocytes in the absence of SUN-1 in the nuclear rim (misalignment in 10 out of 15 gonads in the mutant, in 0 out of 10 in wild type). Bar, 10 µm.

Concomitant with loss of SUN-1, we could not detect ZYG-12 in the ONM in late prophase (Figure 4A). In the absence of a functional SUN-KASH bridge and the proper transduction of cytoskeletal forces to meiocytes, the arrangement and positioning of nuclei is disrupted in the *C. elegans* germ line (K. Zhou *et al.* 2009). In *sun-1(*Δ*158–235)* mutants, instead of a linear alignment of oocytes after cellularization we observed a perturbed distribution (Figure 4B).

We also analyzed protein localization in the *sun-1(*Δ*158–235)*::*gfp* mutants in the presence of endogenous SUN-1. This showed that loss of SUN-1 from the NE in the absence of the cc domains was not rescued in the presence of the endogenous wild-type protein, emphasizing the lack of interaction between the two populations of the protein (Figure S2).

### SUN-1 cc motifs are dispensable for execution of early meiotic prophase I events

**Figure 5:**
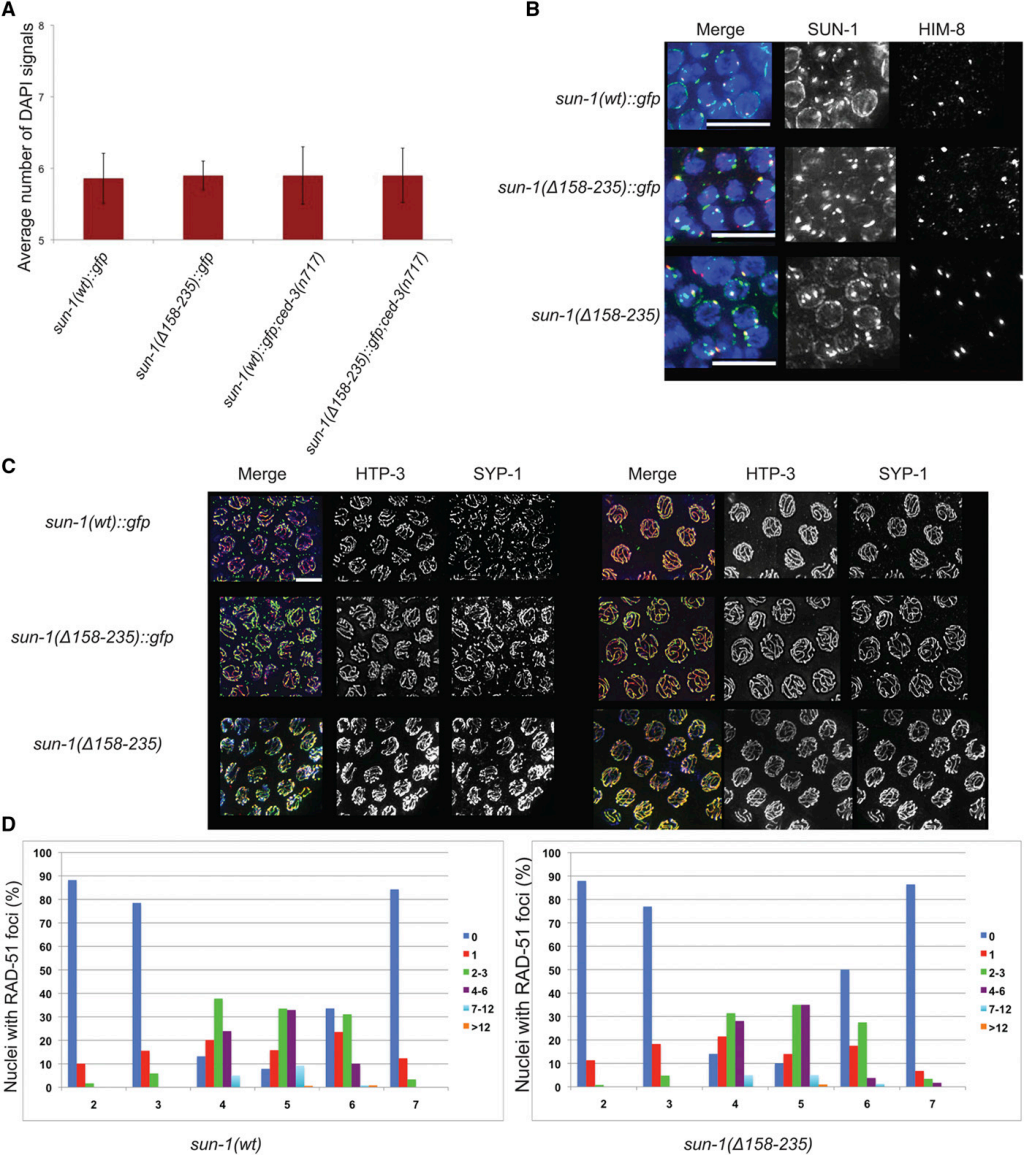
Meiosis in the *sun-1(*Δ*158–235)* germ line. (A) Average number of DAPI signals in −1 diakinesis in the wild type and *sun-1(*Δ*158–235)*::*gfp* mutant in both wild-type and *ced-3(n717)* backgrounds. Bars represent SD. *Psun-1(wt)*::*gfp II*; *sun-1(ok1282) V*, *n* = 22; *Psun-1(*Δ*158-235)*::*gfp II*; *sun-1(ok1282) V*, *n* = 24; *Psun-1(wt)*::*gfp II*; *sun-1(ok1282) V*; *ced-3(n717)*, *n* = 50; *Psun-1(3(n717)*, *n* = 50; *s II*; *sun-1(ok1282) V*; *ced-3(n717)*; *n* = 40. (B) DAPI (blue), SUN-1/GFP (green), and HIM-8 (red) staining in wild type and both tagged and untagged mutant strains. (C) HTP-3 (green) and SYP-1(red) staining of early (left panel) and late (right panel) pachytene nuclei in wild type and mutants. Bar, 10 µm. (D) The dynamics of DSB repair in wild-type (left graph) and mutant (right graph) nuclei. In both cases, *n* = 6.

The formation of six bivalents (homologs connected by crossovers and cohesion) in oocytes results from successful pairing, synapsis, and recombination processes that depend on LINC complex function. As in wild type, six DAPI signals could be detected in the oocytes of both tagged and untagged mutants lacking the cc domains, thus indicating the proper orchestration of meiosis [DAPI signals in diakinesis: wild type = 5.8 ± 0.37, *n* = 31; *sun-1(*Δ*158–235)* = 6.2 ± 1.7, *n* = 42; and Figure 5A]. Apoptosis eliminates defective meiocytes (Gartner *et al.* 2008). However, even in the *ced-3(n717)* apoptosis-deficient background, the cc mutant did not display an increase in achiasmatic chromosomes (Figure 5A). We also investigated homologous pairing, synapsis, and the dynamics of meiotic double-strand-break (DSB) repair in *sun-1(*Δ*158–235)* mutants *vs.* wild type and could not detect any differences (Figure 5, B–D). Staining against HIM-8 (a marker for the *X* chromosome subtelomeric region; Phillips *et al.* 2005, 2009) revealed that pairing was similar in both wild type and mutant (Figure 5B). Our previous studies showed a role for SUN-1 N-terminal modifications in SC polymerization kinetics (Woglar *et al.* 2013). The kinetics of HTP-3 and SYP-1 (chromosome axis and synapsis markers) loading was also similar in both wild type and mutants (Colaiacovo *et al.* 2003). SC polymerization started in the TZ, and chromosomes were fully synapsed by early pachytene (Figure 5C). Also, analysis of the appearance and disappearance of RAD-51 over the entire length of the gonad from the distal mitotic zone until diplotene showed that the dynamics of DSB repair were unaltered in the absence of cc motifs (Figure 5D; Alpi *et al.* 2003).

**Figure 6:**
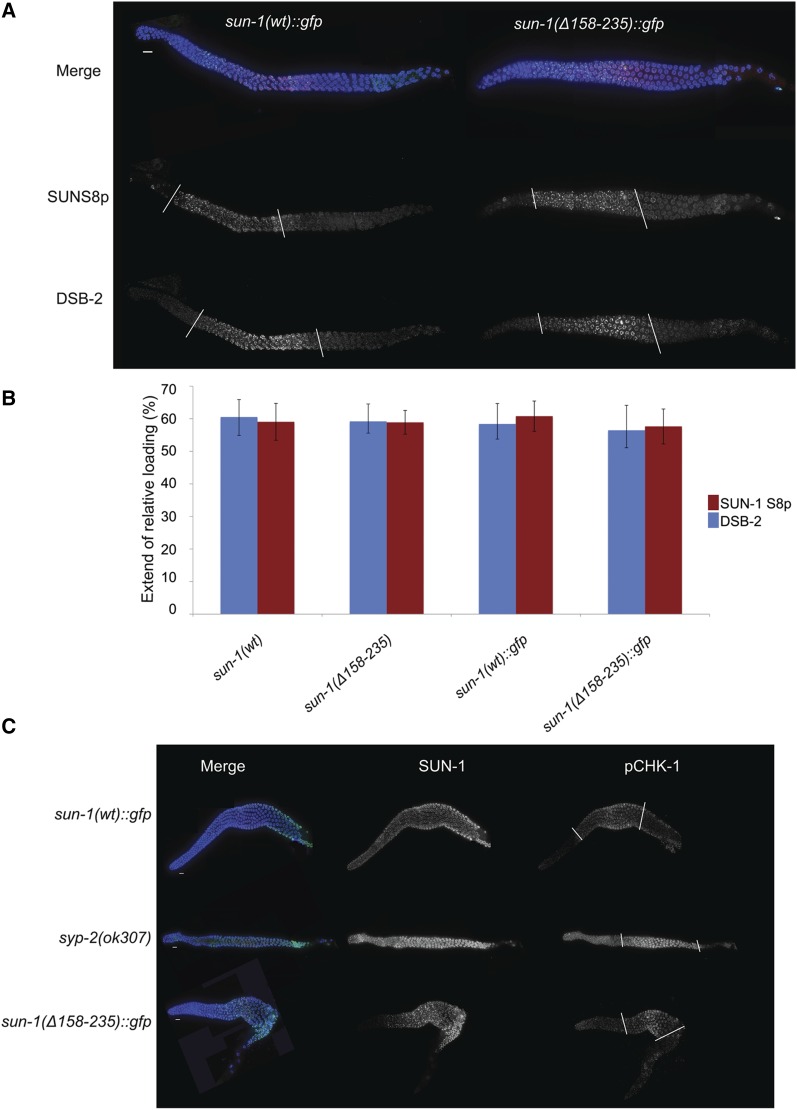
Early meiotic prophase I events progress with wild-type dynamics in the absence of cc motifs. (A) DAPI (blue), SUN-1S8p (green), and DSB-2 (red) staining in wild-type and mutant germ lines; with no differences. White bars mark the zone containing phosphorylated SUN-1 and the zone of DSB-2 localization. (B) Graph showing quantification of the zone positive for phospho-SUN-1S8 and DSB-2. The region positive for these markers was normalized to gonad length from meiotic onset until diplotene. There is no significant difference between wild type and mutant (two-tailed *t*-test, *P >* 0.581). Bars represent SD *Psun-1(wt)*::*gfp II*; *sun-1(ok1282) V*, *n* = 11; *Psun-1(*Δ*158–235)*::*gfp II*; *sun-1(ok1282) V*, *n* = 6; *sun-1(wt)*, *n* = 6; *Psun-1(Δ158–235) II*; *sun-1(ok1282) V*, *n* = 6. (C) Costaining of GFP (to mark SUN-1) and pCHK-1. White bars mark the zone containing pCHK-1. Note the higher signal intensity and extension of the zone containing pCHK-1 until the end of pachytene in the absence of synapsis compared with other genotypes. Bar, 10 µm.

Timely progression through prophase I correlates with the accomplishment of meiotic tasks; such as pairing, synapsis, and generation of a crossover intermediate (of an unknown nature) (Rosu *et al.* 2013; Woglar *et al.* 2013). Previous studies have shown a link between phosphorylation of the N-terminal nucleoplasmic portion of SUN-1 and the time period in which those tasks are accomplished. In the presence of meiotic errors, for instance incomplete synapsis, the zone of phosphorylated SUN-1 is extended, indicating a progression delay (Woglar *et al.* 2013). Staining for phosphorylated SUN-1S8 revealed that the dynamics of phosphorylation and dephosphorylation of this residue are identical in the mutant and wild type (Figure 6, A and B). Staining for two other markers of this region, DSB-2 (DNA-DSB factor; Figure 6, A and B; Rosu *et al.* 2013) and phosphorylated CHK-1 (pCHK-1; Figure 6C; Jaramillo-Lambert *et al.* 2010) also confirmed wild-type progression through meiosis in the absence of cc motifs.

Our results show that despite the reduced amount of SUN-1 in the NE, a functional LINC complex is formed and all early processes of meiotic prophase I were accomplished as in wild type.

Deep sequencing analysis of RNA extracted from the mutant and wild-type worms revealed that the expression of SUN-1 and all known meiotic genes, genes required for early embryonic development, or housekeeping was similar between the two genotypes (Figure S3). The experiment was done in an embryo-deficient background *spe-26(hc138)* to exclude embryonic messenger RNAs (mRNAs) from the analysis. This analysis revealed 17 genes to be differently expressed between wild type and mutant, either up- or downregulated (Table S2). Quantitative PCR, performed with RNA extracted from dissected gonads, did not reproduce different RNA amounts of these ORFs and *sun-1* itself in the mutant (our unpublished results). These data show that the absence of cc regions leads to decreased amounts of SUN-1 in the nuclear rim despite normal expression levels of *sun-1* itself but also other germ line expressed genes. This phenotype becomes most pronounced as meiocytes enter late pachytene/diplotene. However, lack of these domains does not abrogate the formation of the LINC complex and SUN-1 higher-order structures upon meiotic entry. The ability to form SUN-1 aggregates at chromosome end attachments (which support chromosome end mobilization) in the cc mutant strongly supports the notion that formation and function of the LINC complex is independent of SUN-1 oligomerization and prophase I events proceed as in the wild type. SUN-1 cc motifs are therefore required for efficient protein localization to the INM, especially at the later stages of prophase I, in particular during female oogenesis (see below).

### cc motifs are required for SUN-1 retention at the NE rather than for targeting

**Figure 7:**
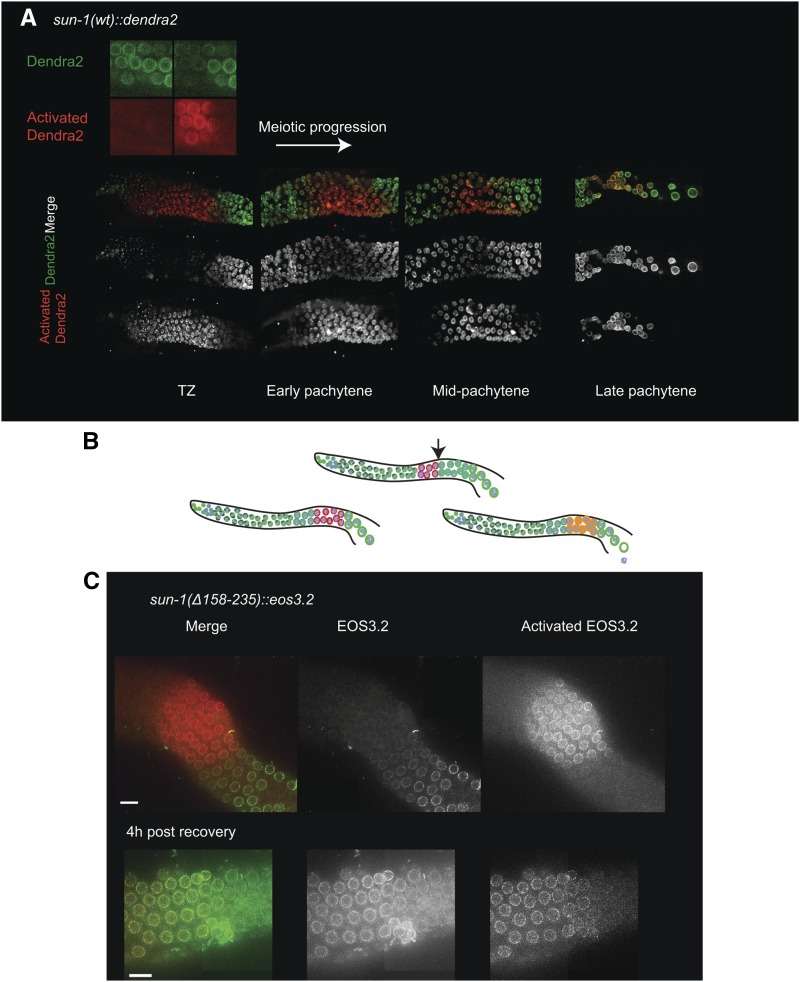
SUN-1 loss is a consequence of defective NE retention. (A) Photo-conversion experiment in *sun-1(wt)*::*dendra2*. Top image, the Dendra2 fluorophore in the activated and nonactivated states; bottom panels, gradual increase in *de novo* SUN-1 incorporation in the TZ, early, mid-, and late pachytene stages. Note the robust incorporation of SUN-1 from midpachytene onwards. (B) Diagrams explaining the photo-conversion experiment with the possible outcomes (also see main text). The arrow indicates the start of the zone with robust incorporation of SUN-1. (C) Top panel reveals the zone of photo-conversion of the Eos 3.2 tag (green to red) which is located prior to robust SUN-1 incorporation at the NE in midpachytene in the *sun-1(Δ158–235)*::*eos3.2* gonad. The bottom panel shows the late pachytene stage at 4 hr post photo-conversion. Note photo-converted and newly inserted SUN-1 molecules at the nuclear rim. Bar, 10 µm.

The amount of SUN-1 localized to the NE increases strongly during prophase and peaks in the cellularized oocyte, suggesting that continuous incorporation of this protein takes place (Figure 3, A and D). Furthermore, photo-conversion experiments using SUN-1 tagged with the photo-convertible fluorophore Dendra2 (full green-to-red photo-conversion) (Gurskaya *et al.* 2006) confirmed our cytological observations and revealed that SUN-1 is continuously inserted into the NE in the wild-type germ line as nuclei progress through meiosis. *De novo* SUN-1 loading to the NE is most prominently seen from midpachytene onwards (Figure 7A).

To find the reason for the lack of SUN-1 in late pachytene/diplotene we tested if cc motifs were required for the targeting of the *de novo* synthesized SUN-1 from midpachytene onwards or whether they were required for efficient retention of SUN-1 in the NE. For this purpose we performed a photo-conversion experiment using a *sun-1(*Δ*158–235)* transgene tagged with the photo-convertible fluorophore Eos 3.2 (M. Zhang *et al.* 2012). We photo-activated SUN-1 in a few rows of nuclei at the midpachytene stage, *i.e.*, the zone showing the most prominent *de novo* loading of SUN-1. Nuclei migrate along the germ line at 1 row/hr (Crittenden *et al.* 2006). Thus, worms were recovered after 4 hr and the distribution of SUN-1 was analyzed. If there were problems in protein targeting, we would be unable to detect newly-incorporated protein at the nuclear rim and the SUN-1 population detected in this zone would consist of only red photo-converted molecules. However, if targeting was normal, we would be able to detect a mixture of activated and nonactivated SUN-1 proteins (Figure 7B). The photo-conversion experiments showed that newly-synthesized wild-type and mutant SUN-1 protein was inserted next to the existing pool (Figure 7C).

When analyzing SUN-1 localization, we noticed blobs of mutant SUN-1 protein in the cytoplasm. This was not observed for wild-type SUN-1 protein and we propose that this is the pool of protein lost from the NE (Figure S4A). Western blot analysis confirmed that the mutant SUN-1 protein amount is reduced, which suggests that once the protein is lost from the NE it is subjected to degradation (Figure S4B).

**Figure 8:**
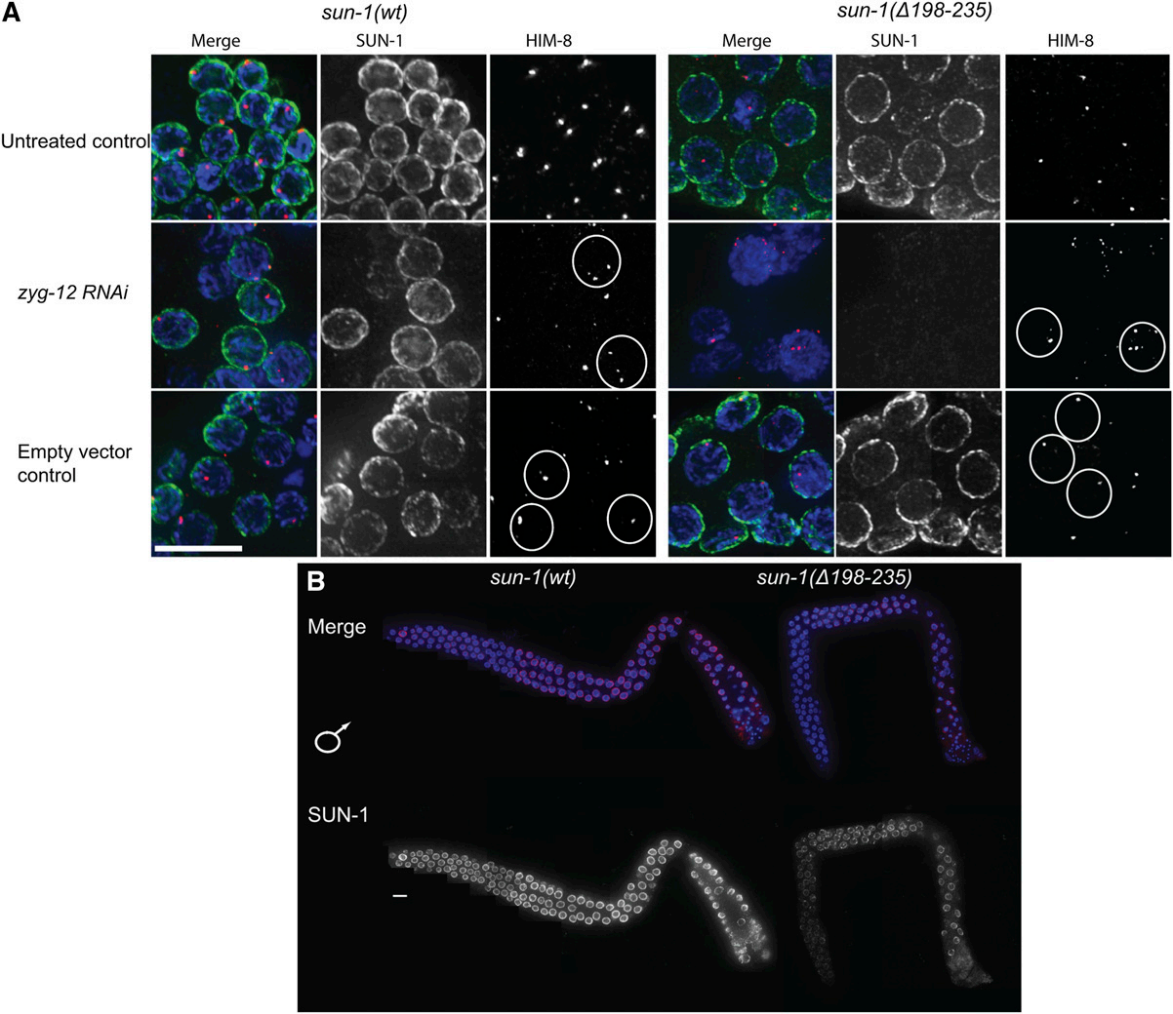
SUN-1 cc regions counterbalance forces on the SUN-KASH bridge. (A) Midpachytene nuclei of *sun-1(wt)* and *Psun-1(*Δ*158–235) II*; *sun-1(ok1282) V* gonads 48 hr post-L4. DAPI (blue), SUN-1(green), and HIM-8 (red) costainings of gonads either treated with *zyg-12* *RNAi*, untreated, or treated with empty vector control. White circles delineate the unpaired *X* chromosomes in case of RNAi treatment and the paired *X* chromosomes when treated with empty vector. Note the severe destruction of the gonad architecture in the absence of both SUN-1 and ZYG-12 in the mutant. (B) Costaining of DAPI (blue) and SUN-1 (red) in L4 gonads (undergoing spermatogenesis) of wild type and *Psun-1(Δ158–235) II*; *sun-1(ok1282) V*. Note the absence of the severe SUN-1 loss in the L4 gonads in the later stages of prophase I in the mutant. Bar, 10 µm.

To address whether nuclear membrane anchorage of cc-deleted SUN-1 was less robust, we depleted the KASH partner ZYG-12 by RNAi. *zyg-12* depletion was assessed in the *zyg-12*::*gfp* line (Figure S5), GFP signal depletion goes in hand with defects in chromosome pairing monitored by HIM-8 (Phillips *et al.* 2005, 2009). We used the consequent pairing defect as a readout for efficient *zyg-12* knockdown since immunostaining against ZYG-12 was challenging. 48 hr post treatment, HIM-8 paired signals were lost in the entire germ line (Figure 8A and Figure S5). In wild type, SUN-1 localization to the NE was not affected (Figure 8A). In contrast, *sun-1(*Δ*158–235)* mutant germ lines were severely affected. Loss of ZYG-12 from the ONM led to a failure to retain SUN-1 in the INM (Figure 8A) and gonads developed a severe sterility phenotype (reminiscent of the *sun-1* deletion mutant phenotypes).

To summarize, our findings are consistent with a model in which cc-deleted SUN-1 is targeted successfully to the INM but fails to be retained efficiently and becomes highly sensitive to strain imposed only from one side of the LINC complex. Once the protein is lost from the NE, it is eventually subjected to degradation.

### The severe loss of SUN-1 in late pachytene/diplotene is specific for oogenesis

We wondered if the prominent loss of SUN-1 in the mutant background was linked to processes prior to oocyte cellularization. To address this we examined nuclei of corresponding stages (late pachytene/diplotene) in the germ line of L4 larvae. Due to the hermaphroditic nature of *C. elegans*, germ cells undergo spermatogenesis in the L4 stage. Here meiocytes do not arrest in diplotene, but instead proceed through the two divisions to produce sperm. During spermatogenesis SUN-1 is not prominently lost from the NE in late pachytene/diplotene in *sun-1(*Δ*158–235)* mutants, despite an overall reduced amount of protein (Figure 8B). We therefore hypothesize that the female germ line-specific loss of SUN-1 prior to diplotene could be linked to processes related to oocyte cellularization.

### C. elegans embryos use the maternally-supplied protein pool of SUN-1

**Figure 9:**
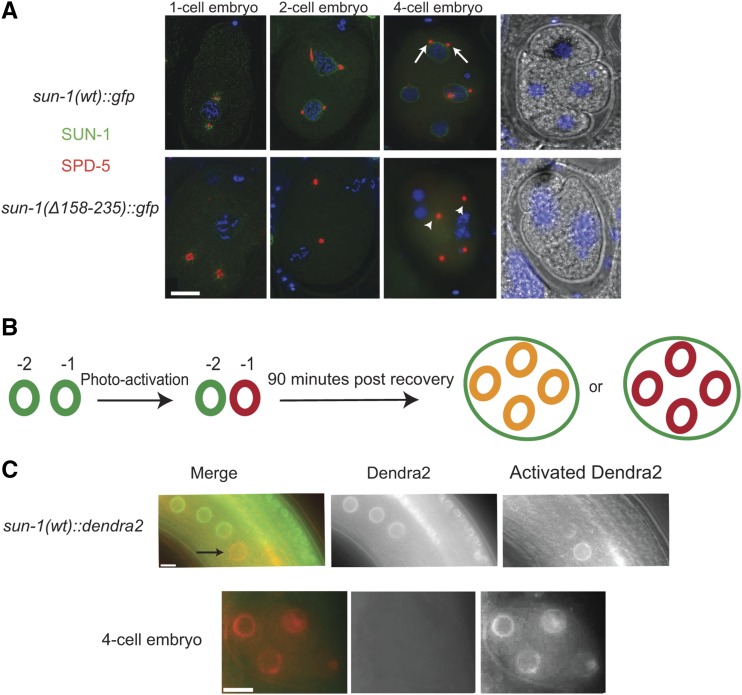
The embryonic SUN-1 pool is maternally supplied. (A) Centrosome detachment in *Psun-1(*Δ*158–235) II*; *sun-1(ok1282) V* mutants. SPD-5 (red) and GFP (green) staining mark the centrosome and SUN-1 in the wild type and mutant. Arrows indicate centrosomes in the vicinity of the NE in the wild type; arrowheads indicate detached centrosomes in the mutant. Note the decrease in SUN-1 signal intensity in mutant embryos. (B) Diagram of the photo-conversion experiment design. If SUN-1 is synthesized in the embryo, then a mixture of red photo-converted protein and green newly-synthesized SUN-1 is expected (resulting in yellow). If embryos exclusively use maternally-supplied SUN-1, a red photo-converted pool is expected. (C) Photo-conversion of SUN-1::Dendra2 in the −1 oocyte (green to red) in the top panel (black arrow). 4-cell embryo at 90 min postrecovery once an embryo has developed from the oocyte (bottom panel). Note the presence of photo-converted SUN-1 protein only. Bar, 10 µm.

While addressing the cause of embryonic death in *sun-1(*Δ*158–235)*, we noticed that SUN-1 was undetectable in these mutants. Costaining of SUN-1 and SPD-5 (Hamill *et al.* 2002) revealed a centrosome detachment phenotype (Figure 9A). In 14 out of 16 early embryos (1–4 cell stage) detached centrosomes were detected. This phenotype has also been observed in the *sun-1(jf18)* mutant that has a dysfunctional SUN-KASH bridge, in which ZYG-12 is not recruited to the ONM (Penkner *et al.* 2007). Centrosome detachment leads to severe aneuploidy and explains the low embryonic viability observed in the mutant.

Failure of SUN-1 retention in the NE, most prominently in late pachytene/diplotene, and lack of SUN-1 in the embryos of the mutants made us ask how SUN-1 is produced in the embryos. We wondered whether SUN-1 protein was deposited in the embryos rather than *sun-1* mRNA, since maternal deposition of SUN-1 has been suggested previously (Fridkin *et al.* 2004). We therefore performed a photo-conversion experiment using wild-type SUN-1 tagged with Dendra2. We photo-activated the SUN-1 population in the −1 oocyte, allowed the animal to recover, and scored for fluorescence in the embryo after 90 min (*i.e.*, 70–90 min postfertilization). If the embryos used the maternal pool of SUN-1 protein, we would expect photo-activated SUN-1 (in red) in the embryos, and if new protein was synthesized in the embryos, a mixture of activated and nonactivated (green) protein would be expected (Figure 9B).

Our result showed that newly-translated SUN-1 is not detectable in early embryos and that embryos exclusively rely on the NE pool of SUN-1 protein maternally supplied to the oocyte (Figure 9C). As a consequence of cc-motif deletion, SUN-1 is lost in oocytes and embryos, and ZYG-12 recruitment to the NE is abrogated. This causes defective centrosome positioning, leading to chromosome mis-segregation in the following mitotic divisions and embryonic lethality.

## Discussion

This study provides further insight into the role of the cc motifs of SUN-domain proteins in LINC complex formation and function. We showed that SUN-1 forms oligomers in the *C. elegans* germ line. Oligomerization depended on the cc motifs but was not a prerequisite for SUN-KASH interaction and the formation of higher-order SUN-1 assemblies. In the absence of SUN-1 oligomerization, a functional LINC complex was formed that could execute mitotic and meiotic functions. Nevertheless, SUN-1 cc motifs and oligomerization were important for SUN-1 retention in the NE. In the absence of self-interaction, SUN-1 was not retained at the INM; this was most pronounced prior to oocyte cellularization. Furthermore, the pool of SUN-1 protein present in early embryogenesis was exclusively derived from maternal protein located at the NE in the oocyte.

### The SUN-1 cc motifs are required for self-interaction

Previous studies showed that SUN proteins form oligomers for which they need an intact SUN domain and adjacent cc motifs (Padmakumar *et al.* 2005; Crisp *et al.* 2006; Q. Wang *et al.* 2006; Lu *et al.* 2008; Sosa *et al.* 2012; Z. Zhou *et al.* 2012; W. Wang *et al.* 2012). Recent *in vitro* studies and crystallization analysis of hSUN2 suggest that SUN proteins have a trimeric configuration that enables them to form a clover-like head, which is a prerequisite for building a functional LINC complex. hSUN2 trimers recruit KASH-domain protein trimers, thereby forming a 3:3 hexameric complex (Sosa *et al.* 2012; W. Wang *et al.* 2012; Z. Zhou *et al.* 2012). Our pull-down assays suggested that in the germ line, SUN-1 proteins always form oligomers irrespective of the meiotic stage. Although it has been suggested that the region extending from the transmembrane domain to the SUN domain could form a single continuous cc (Rothballer *et al.* 2013), our *in vitro* and *in vivo* assays confirmed the work of Minn *et al.* (2009) showing that SUN-1 oligomerization relies on the luminal-predicted cc domains. Surprisingly, and in contrast to published data (Sosa *et al.* 2012; W. Wang *et al.* 2012; Z. Zhou *et al.* 2012), deletion of both cc motifs and thus lack of SUN-1 self-interaction did not impair LINC complex formation and KASH partner recruitment. All early events sustained by SUN-1, such as chromosome movement, synapsis, or DSB repair took place in the deletion mutant with wild-type dynamics.

### The SUN-1 cc domain is required for NE retention

SUN-1 expression in the nuclear rim throughout the entire gonad is significantly weaker in the absence of both cc motifs. Due to a reduced amount of SUN-1 in the nuclear rim, the aggregates appear more prominent and the small aggregates become more obvious. Nevertheless, the amount of SUN-1 protein present at the NE is still sufficient for functionality during meiosis, even under conditions when force is exerted on the SUN-KASH bridge during chromosome movement. Perhaps, at this stage, coalescence of SUN-1 molecules at pairing center sites compensates for the inability for self-interaction. The loss of SUN-1 becomes more prominent when nuclei enter diplotene.

Different studies have revealed that NE localization and membrane targeting of SUN proteins relies on multiple signals that contribute redundantly to their proper localization (Padmakumar *et al.* 2005; Turgay *et al.* 2010; Tapley *et al.* 2011). The precise mechanism by which Cel-SUN-1 is targeted to the INM is unknown. Our work supports a model in which the cc domains are required for Cel-SUN-1 retention in the NE. The use of photo-convertible SUN-1 and the spatiotemporal arrangement of meiocytes allowed us to show that the cc-deleted protein was initially targeted to the NE, but was lost soon after. This was most prominent in late pachytene/diplotene. At this stage of meiocyte development, chromosomes lose contact with the NE and condense to form individual bivalents. The karyosome (the state in which chromatin translocates to the center of the nucleus) is best studied in *Drosophila*; it was shown in this system that the process depends on NHK-1-dependent phosphorylation of the BAF protein, with BAF connecting chromatin to the NE (Lancaster *et al.* 2007). We therefore propose that chromosome detachment from the NE at this stage might contribute to SUN-1 destabilization at the membrane, because SUN-1 oligomerization might support robust NE anchorage to counteract the mechanical strain exerted on the LINC complex from the cytoplasm. SUN proteins are targeted to the INM by a lateral diffusion mechanism (Ungricht *et al.* 2015). Once the protein is targeted, it will be anchored in the INM through binding to the nuclear lamina or other nucleoplasmic proteins on one side and the KASH protein on the other side (Rothballer *et al.* 2013; Link *et al.* 2015). ZYG-12 knockdown via RNAi treatment in the cc mutant led to severe loss of SUN-1, whereas in the wild type SUN-1 localization was not affected. We hypothesize that lack of ZYG-12 and consequently the cytoplasmic anchor leads to dramatic loss of SUN-1 in the mutant, whereas in the wild type SUN-1 oligomerization still supports the retention. During the process of cellularization in diplotene, the cellular and nuclear volumes increase. In wild type, SUN-1 oligomers can resist unbalanced cytoplasmic forces originating from changes in the cytoskeletal architecture when oocytes cellularize; however, disruption of the oligomerization leads to loss of SUN-1 from the NE. Upon loss of NE localization the protein is eventually degraded. Consistently, in the same stage in male gametogenesis (late pachytene/diplotene) SUN-1 is not lost from the NE.

The cc-deleted strains allowed us to ask whether gene expression was altered upon loss of protein, as had been observed in SUN-1 mutant mice (Chi *et al.* 2009). Premature loss of SUN-1 did not alter gene expression, in particular *sun-1* expression itself.

### Embryogenesis relies on the maternal pool of SUN-1

*C. elegans* embryos use the maternal supply of SUN-1 protein, thus emphasizing the importance of *de novo* SUN-1 incorporation into the NE during prophase I, especially from midpachytene onwards. Current models suggest that NE proteins are retracted to the ER during mitosis and that this pool is reused for NE reassembly after mitosis (Wandke and Kutay 2013). The failure of SUN-1 NE retention leads to a lack of SUN-1 in both the oocyte and the developing embryo. Therefore, SUN-1 cc-mutant embryos display defects in centrosome attachment due to the absence of a functional SUN-KASH bridge (Fridkin *et al.* 2004; Penkner *et al.* 2007; Meyerzon *et al.* 2009).

Altogether we show that in *C. elegans*, formation of a functional SUN-KASH complex in the germ line does not depend on an oligomeric conformation of SUN-1. In the absence of SUN-1 self-interaction, functional LINC complexes and SUN-1 higher-order structures are formed at meiotic entry. Nevertheless, cc domains are required for SUN-1 retention in nuclear membranes. This is of the utmost importance for the development of healthy zygotes because the maternal pool of SUN-1 that is necessary for embryonic development is provided through the nuclear membrane of the oocyte.

## 
